# Direct Electrophysiological Mapping of Shape-Induced Affective Perception

**DOI:** 10.1155/2018/9795013

**Published:** 2018-08-02

**Authors:** Yingli Li, Qingguo Ding, Yuancun Zhao, Yanan Bu, Xiaoyan Tang, Peiguo Wang, Genhua Zhang, Mengling Chen, Pei Liang

**Affiliations:** ^1^Sensory Cognition and Design Institute, Changshu Institute of Technology, Changshu, China; ^2^The School of Education, Soochow University, Soochow, China; ^3^The No. 2 Peoples' Hospital of Changshu, Changshu, China

## Abstract

Visual information may convey different affective valences and induce our brain into different affective perceptions. Many studies have found that unpleasant stimuli could produce stronger emotional effects than pleasant stimuli could. Although there has been a notion that triangle is perceived as negative and circle as positive, there has been no systematic study to map the degrees of valence of shapes with different affective perceptions. Here, we employed four shapes (ellipse, triangle, and line-drawn happy and angry faces) to investigate the behavior and electrophysiological responses, in order to systematically study shape-induced affective perception. The reaction time delay and the event-related potential (ERP), particularly the early ERP component, were applied to find the associations with different affective perceptions. Our behavioral results showed that reaction time for angry face was significantly shorter than those for the other three types of stimuli (*p* < 0.05). In the ERP results, P1, N1, P2, and N2 amplitudes for angry face were significantly larger than those for happy face. Similarly, P1, N1, P2, and N2 amplitudes for triangle were significantly larger than those for ellipse. Particularly, P1 amplitude in the parietal lobe for angry face was the strongest, followed by happy face, triangle, and ellipse. Hence, this work found distinct electrophysiological evidence to map the shape-induced affective perception. It supports the hypothesis that affective strain would induce larger amplitude than affective ease does and strong affective stimuli induce larger amplitude than mild affective stimuli do.

## 1. Introduction

Threat detection from visual perception has been developed for our survival over the time span of the biological evolution. From conscious to subconscious levels, our brain has been tuned to be sensitive to all kinds of affective information with different degrees of positive and negative values. For instance, facial expressions such as a happy face and an angry face are consciously perceived as positive and negative in social interaction [1]. Some isolated schematic facial expressions such as V-shaped downward eyebrow configuration have been rated to be more negative and arousing than upside-down patterns [2]. Even a simple shape, which is similar to the geometric configuration of the face in angry expression, is perceived as threatening. In 2006, Aronoff reviewed how humans recognize angry and happy emotions in people, places, and objects. He demonstrated that it is the geometrical patterns, such as diagonal and angular configurations, rather than actual facial features that conveyed the message of threats, while round and curved shapes were linked to warmth [3]. Growing evidence suggests that the underlying geometry of a visual image might serve as an effective vehicle for conveying the affective meaning of a scene or of an object. A recent study has demonstrated that downward triangle is perceived as negative and circle as positive, and their emotional meanings can be activated automatically, as shown at both behavioral and electrophysiological levels [4]. Hence, the questions arise: From complex faces to simple geometric shapes, is there any shared cognitive processing? What is the perception advantage of shapes developed in our brain?

Kahneman hypothesized that information in the human brain may be processed in different psychological statuses, such as cognitive “ease” and cognitive “strain” [5]. In each status, the information flow might be mediated by different networks of the brain. Motivated from Kahneman's theory, we further proposed that visual information might induce *affective ease* or *affective strain* in our brain. Such affective status might modify the brain's processing of information flow and thereby influence our perception of the environment. For instance, it has been observed that detecting an angry face among happy faces is faster and more accurate in contrast to a happy amid angry ones [6]. A similar effect has been found not only in faces but also in shapes. Triangles are more easily detected among circles than the reverse [7]. Negative things elicit a more rapid and more prominent response than nonnegative events do [8]. Moreover, the emotional valences of the geometrical figures have been demonstrated to impact even cross-sensory perceptions such as taste. For instance, a circular shape may enhance sweetness sensitivity [9], and this effect is invariant across different cultures [10]. All these findings support that different affective states may influence the neural information processing and the behavior pattern. Few fMRI studies have elucidated that downward-pointing triangles activated the same neural circuitry known to facilitate the processing of realistic, contextual threatening stimuli [11]. Bilateral amygdales were more strongly activated by angular objects than by curved ones [12]. However, the neural basis of such subtle impact of geometrical figures on perception remains unclear still. Hence, our present study is aimed at exploring the human brain's reaction to “positive” and “negative” geometrical figures and finding the evidence of electrophysiological mapping of shape-induced affective ease and affective strain.

The ERP is a powerful electrophysiological technique for measuring brain activation signals, with a time resolution accurate down to milliseconds [13]. The literature suggests that early ERP components are sensitive to emotional stimuli, and the right hemisphere plays a critical role in emotion processing. Some local brain regions such as the frontal lobe and the parietal lobe are especially sensitive to emotional pressure [14]. Recent studies find that the target detection sensitivity for a negative emotional stimulus was higher than that for a neutral stimulus. ERP revealed that high-intensity anger expressions elicited larger P3a and late positive potential amplitudes relative to prototypical anger expressions for power-motivated individuals [15]. N170 response to facial expressions is modulated by the affective congruency between the emotional expression and preceding affective pictures [16]. Positive emotions evoke N170 significantly earlier than negative emotions do, and the amplitude evoked by fearful faces was larger than that evoked by neutral or surprised faces [17]. The greater the affective distance of a target, the larger the late potential. In the present study, we chose six early ERP components (N1, P1, N2, P2, N3, and P3) to investigate the electrophysiological correlates of cognitive status.

In the present study, we hypothesized that the two groups of stimuli (positive versus negative) would be differentiable in early ERP components, despite that facial stimuli might elicit stronger brain activation than geometrical stimuli might. To compare directly the perception of simple geometric shapes and faces, which are with different degrees of affective values, a comparison of two target shapes (i.e., an ellipse and a triangle) and two emotional shapes (i.e., a line-drawn happy face and an angry face) was carried out. Moreover, the present study deals with a particular process within the emotion reaction: attention to affective stimuli. Since shapes provide very abstract information, it may influence our behavior subconsciously. We hypothesize that the angry face and triangle may lead the brain into a similar perception of affective strain, while the happy face and ellipse will take the brain to a perception of affective ease. The present study is to test this hypothesis that simple geometric forms convey emotion and that this perception does not require explicit judgment. In this study, the following questions will be addressed: (1) Do brain EEG signals elicit different patterns for affective ease and affective strain? (2) If so, what is the difference between these two groups? (3) Does the brain EEG signal behave similarly for the stimulus from the same group? (4) Can we generalize the stimulation types and predict our brain response? All these questions will be answered and discussed in the end.

## 2. Methods

### 2.1. Participants

Twenty randomly selected college students (10 males and 10 females, averaged age = 23.11, SD = 1.53) participated in the present study. All participants were right-handed, with normal or normal-after-correction vision. They were well explained about the details of their performance. They have all agreed and signed on the written informed consent declaration to volunteer as subjects in these experiments. The study was approved by the Ethics Committee of Changshu Institute of Technology, according to the National Ethics Guidelines.

### 2.2. Materials

**Figure 1:**
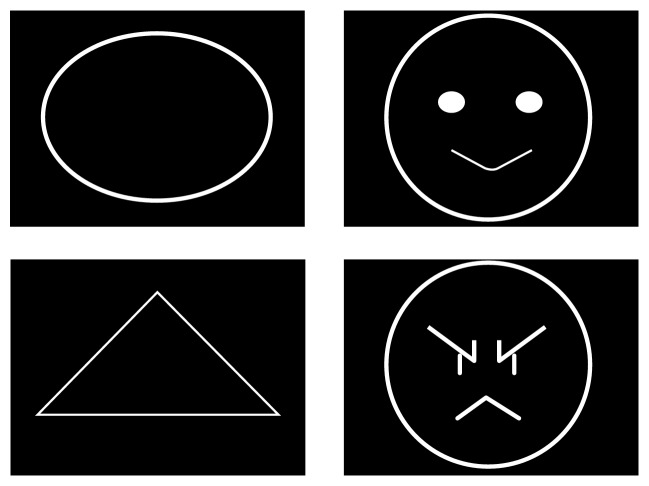
The left side illustrates ellipse and triangle as the target stimuli, and the right side shows a line-drawn happy face and an angry face as stronger emotional stimuli.

Two target shapes (i.e., ellipse and triangle) and two emotional shapes (i.e., smile and angry faces) for comparison were designed (see Figure 1). The four shapes (with a diameter of about 20 cm) were programmed via E-Prime 2.0 to present on a computer screen in a random sequence, with each shape repeated for 100 times, resulting in 400 trials in total. Each shape lasted for 1.5 s, interpolated by a 2 s interval. A practice section consisting of 12 trials (3 trials for each shape) was also programmed in the same way. Participants were required to identify each shape and press the corresponding key on a keyboard by using the index and middle fingers of both hands (“D” represents ellipse, “F” represents triangle, “J” represents happy face, and “K” represents angry face) as quickly and as accurately as possible.

### 2.3. EEG Recording

After signing a consent form, participants were seated in front of a computer screen in a sound-proof chamber and fitted with a 32-channel Neuroscan electrode cap. All electrodes were positioned in accordance to the International 10-20 System (Binnie, Dekker, Smit, and Van der Linden, 1982) and referenced to CZ (central cortex) during recording. An EOG (electrooculogram) was also recorded from electrodes placed above and below each eye. Electrode impendence was maintained below 5 k*Ω* with a sampling rate of 500 Hz and a 0.15–50 Hz band-pass filter.

Participants were trained during the practice section as long as they needed to be familiarized with the key-pressing pattern before the formal test. They were required to keep their body as still as possible during the formal test, in which the EEG signals were concurrently recorded.

### 2.4. EEG Data Analysis

EEG data were analyzed using standard off-line procedures in BrainVision Analyzer software (Brain Products GmbH, Germany). After eye blink correction, other artifacts (i.e., epochs with EEG power exceeding ±100 microvolts) were removed from the EEG data, and 96.6% of the original EEG data were retained. Subsequently, these artifact-free data were segmented into 1000 ms epochs, baseline-corrected with a 100 ms prestimulus interval, and averaged, respectively, for the four types of stimuli. Based on the literature suggesting that early ERP components are sensitive to emotional processing [18, 19], peak amplitudes were computed for N1 (50 ms–150 ms), P1 (50 ms–150 ms), N2 (150 ms–250 ms), P2 (150 ms–250 ms), N3 (250 ms–350 ms), and P3 (250 ms–350 ms). For our research interest, peak amplitudes of the abovementioned six ERP components in eight selected electrodes (F3, F4, P3, P4, T7, T8, O1, and O2) were computed to represent frontal, parietal, temporal, and occipital lobes in both left and right hemispheres.

## 3. Results

### 3.1. Behavioral Data

A single-factor repeated-measures ANOVA with type as the independent variable and accuracy as the dependent variable showed no significant result. The reaction time of the stimuli for triangle, ellipse, angry face, and smiling face is 376 ± 102, 380 ± 108, 366 ± 104, and 379 ± 100 milliseconds, respectively. The same ANOVA with reaction time as the dependent variable showed a significant main effect for type: *F*(3, 54) = 3.62, *p* < 0.05. Post hoc analysis revealed that reaction time for angry face was significantly shorter than those for the other three types of stimuli (*p* < 0.05). Although the average reaction time for triangle is shorter than that for ellipse, we did not find significance of difference in behavior level.

### 3.2. ERP Data

**Figure 2:**
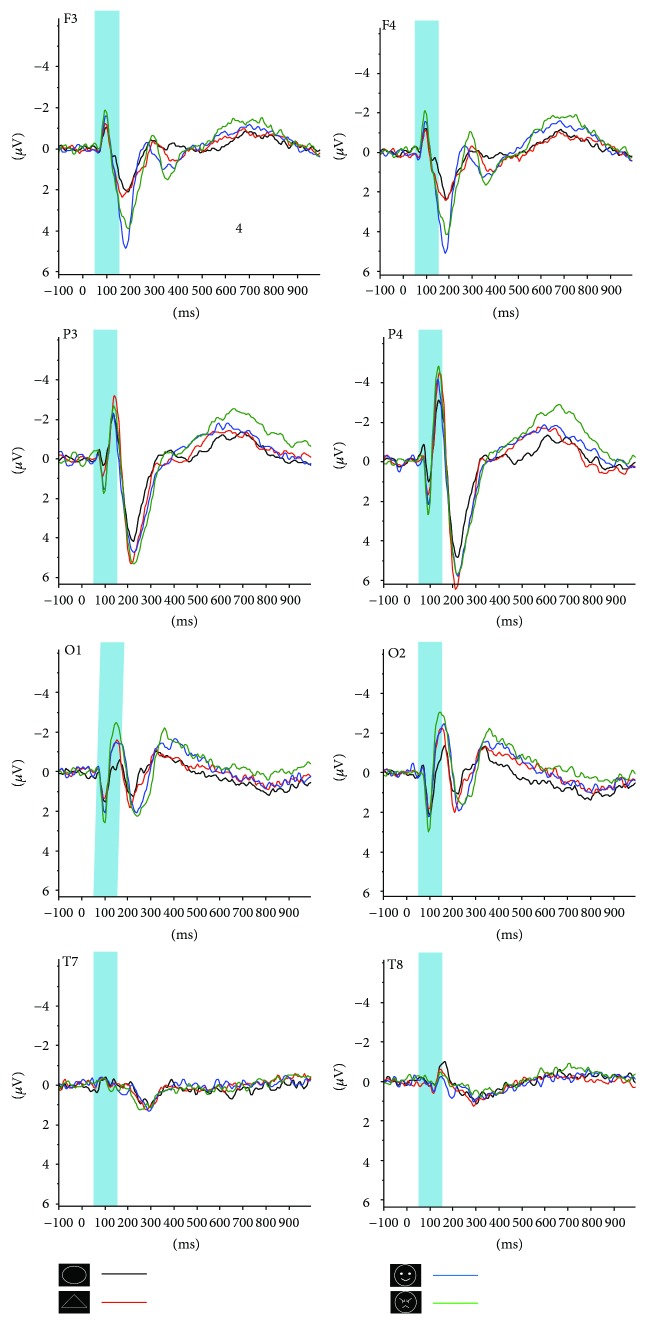
F3, F4, P3, P4, O1, O2, T7, and T8 are the examples of grand averaged waveforms of the left and right frontal, parietal, occipital, and temporal lobes, respectively. Black and red and blue and green lines represent the responses of the ellipse and triangle and smiling and angry faces as stimuli, respectively. The blue bars represent the time windows for ERP N1 component analysis.

**Figure 3:**
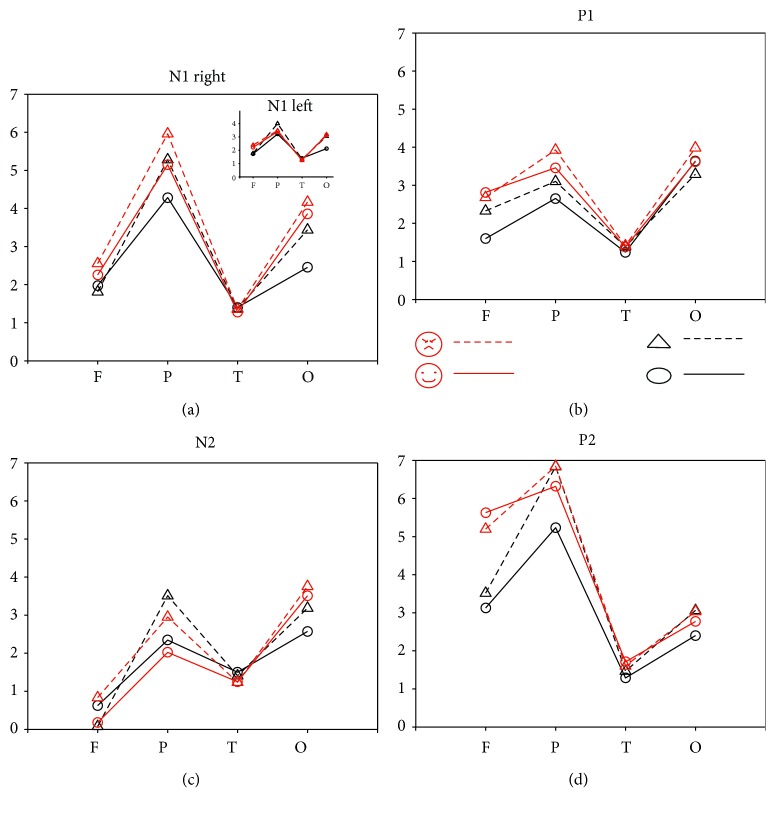
(a) N1 amplitude differences among the four types of stimuli; (b) P1 amplitude differences among the four types of stimuli; (c) N2 amplitude differences among the four types of stimuli; (d) P2 amplitude differences among the four types of stimuli. Note: LF = left frontal; RF = right frontal; LP = left parietal; RP = right parietal; LT = left temporal; RT = right temporal; LO = left occipital; RO = right occipital.

2 (hemisphere: left versus right hemisphere) × 4 (lobe: frontal, parietal, temporal, and occipital) × 4 (type: ellipse, triangle, smiling face, and angry face) within-group repeated-measures ANOVAs were done separately for the six early ERP components (N1, P1, N2, P2, N3, and P3).  Figure 2 depicts the examples of average waveform of the ERP induced with different stimuli from the frontal, parietal, occipital, and temporal lobes, respectively. The averaged response amplitudes for the four early components of the four lobes are illustrated in Figure 3.

#### 3.2.1. N1

A significant main effect was found for type (*F*(3, 54) = 5.85, *p* < 0.01). Post hoc analysis showed that N1 amplitude for angry face was significantly larger than that for happy face (*p* < 0.05), and N1 amplitude for triangle was significantly larger than that for ellipse (*p* < 0.05). These results suggested that the “cognitive strain” group (i.e., angry face and triangle) induced larger response amplitude than the “cognitive ease” group did (i.e., happy face and ellipse). Lobe responses vary significantly (*F*(3, 54) = 10.22, *p* < 0.001). N1 amplitude in the temporal lobe was significantly smaller than that in the parietal lobe (post hoc, *p* < 0.01) and that in the occipital lobe (post hoc, *p* < 0.01). The right hemisphere responded significantly stronger than the left hemisphere did (*F*(1, 18) = 14.03, *p* < 0.01).

Significant interaction effects for type × lobe (*F*(9, 162) = 3.28, *p* < 0.01), type × hemisphere (*F*(3, 54) = 2.94, *p* < 0.05), lobe × hemisphere (*F*(3, 54) = 7.69, *p* < 0.001), and type × lobe × hemisphere (*F*(9, 162) = 2.66, *p* < 0.01) were found as well. Follow-up Bonferroni-corrected paired *t*-tests for the three-way interaction effect showed that N1 amplitude for happy face was significantly larger than that for ellipse (*p* < 0.05) in the left frontal lobe; N1 amplitude for angry face was significantly larger than that for triangle (*p* < 0.05) in the right frontal lobe; N1 amplitude for ellipse was significantly smaller than that for triangle (*p* < 0.05) and that for happy face (*p* < 0.05), as well as that for angry face (*p* < 0.01) in the right parietal lobe; N1 amplitude for ellipse was significantly smaller than that for triangle (*p* < 0.05) and that for happy face (*p* < 0.5) in the left occipital lobe; and N1 amplitude for ellipse was significantly smaller than that for happy face (*p* < 0.01) and angry face (*p* < 0.05) in the right occipital lobe.

#### 3.2.2. P1

Significant main effects were found for type (*F*(3, 54) = 7.73, *p* < 0.001; post hoc analysis showed that P1 amplitude for angry face was significantly larger than that for ellipse (*p* < 0.05) and that for triangle (*p* < 0.05)), lobe (*F*(3, 54) = 6.44, *p* < 0.01; post hoc analysis showed that P1 amplitude in the temporal lobe was significantly smaller than those in the other lobes (*p* < 0.05)), and hemisphere (*F*(1, 18) = 9.62, *p* < 0.001; P1 amplitude in the right hemisphere was significantly larger than that in the left hemisphere (*p* < 0.01)).

A significant interaction effect for type × lobe was also found (*F*(9, 162) = 3.07, *p* < 0.01). Follow-up Bonferroni-corrected paired *t*-tests showed that P1 amplitude for ellipse was significantly smaller than that for happy face (*p* < 0.5) and that for angry face (*p* < 0.5) over the frontal lobe; P1 amplitude for angry face was significantly larger than that for ellipse (*p* < 0.01) and triangle (*p* > 0.05) over the parietal lobe.

#### 3.2.3. N2

Significant main effects were found for lobe (*F*(3, 54) = 10.66, *p* < 0.001; N2 amplitudes in the occipital lobe and the parietal lobe were significantly larger than those in the frontal lobe (*p* < 0.01) and temporal lobe (*p* < 0.05)) and hemisphere (*F*(1, 18) = 5.50, *p* < 0.001; N2 amplitude in the right hemisphere was significantly larger than that in the left hemisphere).

A significant interaction effect for type × lobe (*F*(9, 162) = 8.27, *p* < 0.001) was also found. Follow-up Bonferroni-corrected paired *t*-tests for this interaction effect showed that N2 amplitude for angry face was significantly smaller than that for ellipse (*p* < 0.01) and that for triangle (*p* < 0.05), as well as that for happy face (*p* < 0.01) in the frontal lobe; N2 amplitude for angry face was significantly larger than that for happy face (*p* < 0.05) and N2 amplitude for triangle was significantly larger than that for ellipse (*p* < 0.01) in the parietal lobe; N2 amplitude for angry face was significantly larger than that for happy face (*p* < 0.05) in the occipital lobe.

#### 3.2.4. P2

Significant main effects were found for type (*F*(3, 54) = 12.69, *p* < 0.001; P2 amplitude for ellipse was significantly smaller than those for other figures (*p* < 0.01)) and lobe (*F*(3, 54) = 26.61, *p* < 0.001; P2 amplitude in the temporal lobe was significantly smaller than that in the frontal lobe (*p* < 0.05) and that in the parietal lobe (*p* < 0.001), as well as that in the occipital lobe (*p* < 0.001)).

A significant interaction effect for type × lobe (*F*(9, 162) = 6.27, *p* < 0.001) was also found. Follow-up Bonferroni-corrected paired *t*-tests for this interaction effect showed that P2 amplitudes for happy and angry face were significantly larger than those for ellipse and triangle (*p* < 0.001) in the frontal lobe; P2 amplitude for triangle was significantly larger than that for ellipse (*p* < 0.01) in the parietal lobe.

#### 3.2.5. N3

A significant main effect for lobe was found: *F*(3, 54) = 8.75, *p* < 0.001. N3 amplitude in the occipital lobe was significantly larger than those in other lobes (*p* < 0.01).

#### 3.2.6. P3

A significant main effect for lobe was found: *F*(3, 54) = 4.38, *p* < 0.01. P3 amplitude in the parietal lobe was significantly larger than that in the occipital lobe (*p* < 0.01).

## 4. Discussion

The aim of this study was to investigate the neural responses to different line-drawn configurations, which may induce the brain into different affective perceptions. Our hypothesis is that the circular shape leads the brain into mild “affective ease,” which means the subject feels relaxed and comfortable subconsciously, whereas the angular shape induces mild “affective strain.” Likewise, the happy and angry faces make the subject feel more relaxed or stressed consciously. The results obtained here support our hypothesis. We observed that the reaction time with the angry face is significantly shorter than that with the happy face, and the response amplitudes of P1, N1, P2, and N2 with angry face are significantly larger than those with happy face (*p* < 0.05). On the other hand, subjects respond to triangle with significantly larger amplitudes than to ellipse (*p* < 0.05). However, the reaction time for triangle is not significantly shorter than that for ellipse. Among the four types of stimuli, the early component P1 amplitude in the parietal lobe for angry face is the strongest, followed by happy face, triangle, and ellipse. The overall response of the right hemisphere is stronger than that of the left one.

For the behavior findings, our observation is consistent with the previous literature reports [3, 12, 20, 21]. The negative stimuli (affective strain group) elicit a faster and stronger response than the positive stimuli do (affective ease group). It is worthy of mentioning that negative stimuli evoked a stronger response in early EPR components than did positive stimuli, which was mostly mediated by the parietal and occipital lobes, as shown in N1, P1, and N2 components. It is known that the parietal and occipital lobes play critical roles in visual information processing, with the occipital lobe mediating the primary coding of visual configuration and the parietal lobe further supporting detailed analysis of spatial organization of visual stimuli. Early sensitivity of the two lobes to affective stimuli suggests that the emotional meaning of visual stimuli can be aroused in a very early stage of information processing. This early processing of emotional signals (i.e., identifying “threat” or “nonthreat”) is likely to help humans survive in a complex environment. Moreover, the timescale of the early response of visual affective stimuli also matches with previously published research results [22].

In general, the faces have more complex information and strong affective expression and could induce strong arousal and affective values [23]. The simple geometric shapes are usually treated as much less affective or almost neutral stimuli. However, it has been shown that angular shapes may activate fear and be crucial to processing the threat cues and negative emotion and thus modulate the behavior and performance in real life [11]. Even at the peripheral level, research has demonstrated that triangle and circle could modulate the skin conductance resistance and the startle reflex differently [24]. Here, we could expect that the four types of stimuli applied in our experiments may induce the brain into different degrees of affective strain and affective ease. Our data here matches our expectation. The affective strain group induces a stronger response amplitude than the ease group does (N1, P1, N2, and P2). Within the strain group, angry face induces a larger amplitude than triangle does (N1 right frontal lobe, P1 parietal lobe, and N2 frontal lobe). Within the ease group, happy face induces a stronger response than ellipse does (N1 left frontal, right parietal, left and right occipital, and P1 and N2 frontal lobes). These results indicate that at the early stage, the brain responds to the variant affective visual stimuli differently. The prominent activities of N1, P1, N2, and P2 have significant main effects of different shape-induced degrees of cognitive ease and cognitive strain.

Interestingly, the frontal lobe was more sensitive to facial figures than to geometrical figures (i.e., as shown in N1, P1, and P2 components). This result is consistent with the literature showing that the frontal lobe, especially the lateral inferior prefrontal lobe, is the “social part” of the human brain, which deals with social relationships and functions critically in empathy. A recent review of ERP studies has demonstrated that affective stimulus factors primarily modulate ERP component amplitude [8]. Affective ERPs have been linked to attention orientation for unpleasant pictures at earlier components. Many face ERP studies have shown that emotion facial expressions elicit an early fronto-central positive shift, ranging from 120 to 180 ms poststimulus [25]. Another recent EEG study has shown that emotional facial expression evokes faster attention orientation, but weaker affective neural activity and behavioral responses, compared to that when exposed to emotional scenes [26]. Our data are different from this study, as we did not use the real human face and real scene as stimuli, but simple lines and curves. Thus, our stimuli are much simpler, abstract, and mild compared with the real face pictures. This point is consistent with the finding from Rossi et al. that photographic but not line-drawn faces show early perceptual neural sensitivity [27]. Moreover, Salgado-Montejo et al. found that facial gestures that are associated with specific emotions can be captured by simple shapes and lines [28]. Hence, different types and different intensities of visually elicited emotions may be mapped with different patterns of early responses. For instance, positive emotional faces evoked N170 significantly earlier than did negative emotional faces and the amplitude of fearful faces was larger than that of neutral or surprised faces [17]. Within the same type of emotion, different intensities of angry facial expression lead to different response patterns; higher intensity induces larger P3 and late positive potential [15]. Consistent along the above lines, in our study, different degrees of affective states evoke different response patterns. Affective strain induces larger amplitude of early ERP than affective ease does, and higher intensity of affective states evokes larger amplitude of early ERP than lower intensity of affective states does.

Studies on simple geometric shapes have received more attention recently in behavior and neural physiological research. Using circle and downward triangle as affective priming, Wang and Zhang found a typical effect of affective congruency in the task of face and word analysis [4]. Consistent with previous studies, here we show that triangle is perceived as “affective strain” and evokes larger amplitudes than ellipse does which is perceived as “affective ease.” Therefore, our study has extended previous studies and has shown directly the event-related brain potentials with simple line-drawn shapes and faces. It may suggest that the perception advantage of shapes might be activated from conscious to subconscious levels.

Regarding the neural network of emotion processing, the amygdala, nucleus accumbens, hypothalamus, hippocampus, insula, cingulate cortex, and orbitofrontal cortex have been suggested to be involved [29]. Due to the vague spatial information of EEG, the signals from parietal and occipital channels are most reliable for visually elicited human emotion encoding and classification [14]. In our study, we observed that significant effects of angry and happy faces are registered in frontal, parietal, and occipital lobes. The other significant effects of triangle and ellipse are found in the parietal lobe. Hence, our data support the hypothesis that different degrees of affective states could be mapped with different patterns of neural activities.

Earlier studies showed that basic facial expressions can be processed very rapidly which also includes emotional information processing [17]. Neutral and positive emotions like happiness and pleasant surprise evoked N170 more rapidly than did negative emotions like fear, sadness, and disgust. It has been proposed that a subcortical pathway conveys information more rapidly to various ventral pathways than does the N170 latency. For the negative emotion, the subcortical feedback loop activates a larger underlying neuronal network. In the literature, no subcortical sources have been shown to be active before 140 ms (early processing periods) most likely due to the insensitivity of ERP methods to deep and transient sources. Later activation of subcortical sources (after 320 ms) has been attributed to the extensive spatial and temporal activation at such later time periods. It has also been shown earlier that middle and superior temporal regions are activated in the intermediate time periods (140–400 ms). These areas are particularly sensitive for processing of human and facial expressions. Earlier ERP and clinical studies further suggest the activation of the right lentiform nucleus along with basal ganglia for angry, sad, and neutral faces. Moreover, amygdala activation is invisible to ERP methods. Clinical studies on patients with cerebral injuries have proposed inferior frontal and ventral areas to process recognition in humans. It has been suggested elsewhere that separate recognition of fear, anger, and disgust involves separate neural systems altogether. To localize the accurate areas in the brain for processing of shapes, we need to apply fMRI studies as the next step of investigation in the future.

In short, this study has shown directly the electrophysiological mapping of the brain with different degrees of affective perception induced by visual shapes. It would be interesting to add more parameters to the shapes, such as asymmetry and complexity, to systematically study the influence of shape on inducing different cognitive perceptions. Since shapes are in general more abstract and context-free, this study will help to understand the configuration-induced affective cognition and the related cross-modal sensory integration.

## 5. Conclusions

To conclude, our results provide the first neurophysiological evidence that two geometrical figures which are opposite in emotional valence can be identified as “threat' and “nonthreat” in the human mind. Consistent with our hypothesis, happy and angry faces, as commonly perceived strong emotional signals in social interaction, aroused stronger ERP amplitudes than the two target geometrical figures did (i.e., ellipse and triangle). Importantly, ellipse and triangle were found to arouse similar ERP responses to happy and angry faces (i.e., as shown in N1 and N2 components), respectively. Our ERP data showed that the right hemisphere was more sensitive to emotional stimuli than the left hemisphere, which is consistent with the emotional role associated with the right hemisphere, as reported in previous studies.

## Data Availability

The data used to support the findings of this study are available from the corresponding author upon request.
